# Telomere Length Is Determined by Intrinsic Factors and Is Shortened During Drought Years in *Gallotia galloti*

**DOI:** 10.1002/ece3.73549

**Published:** 2026-05-05

**Authors:** Edward Gilbert, Megan L. Power, Annika Wolberg, Rodrigo Megía‐Palma, Anamarija Žagar, Marta López‐Darias, Miguel A. Carretero, Nina Serén, Pedro Beltran‐Alvarez, Katharina C. Wollenberg Valero

**Affiliations:** ^1^ School of Natural Sciences, The University of Hull Hull UK; ^2^ Energy and Environment Institute, The University of Hull Hull UK; ^3^ School of Biology and Environmental Science, University College Dublin Dublin 4 Ireland; ^4^ Department of Biological Sciences Wellesley College Wellesley Massachusetts USA; ^5^ Department of Biodiversity, Ecology and Evolution School of Biological Sciences, Complutense University of Madrid (UCM) Madrid Spain; ^6^ BIOPOLIS Program in Genomics, Biodiversity and Land Planning CIBIO, Campus de Vairão Vairão Portugal; ^7^ CIBIO Research Centre in Biodiversity and Genetic Resources, InBIO Universidade do Porto Campus de Vairão Vairão Portugal; ^8^ National Institute of Biology Ljubljana Slovenia; ^9^ Biotechnical Faculty, University of Ljubljana Ljubljana Slovenia; ^10^ Delegación del CSIC en Canarias Tenerife Canary Islands Spain; ^11^ Departamento de Biologia Faculdade de Ciências, Universidade do Porto Porto Portugal; ^12^ Biomedical Institute for Multimorbidity Centre for Biomedicine, Hull York Medical School, The University of Hull Hull UK; ^13^ Conway Institute University College Dublin Dublin 4 Ireland

**Keywords:** climate change, ectotherms, model partitioning, oxidative stress, telomeres

## Abstract

Telomeres have emerged as important indicators of organismal longevity and population health; however, our understanding of their dynamics in ectotherms remains incomplete. Here, we investigated variables influencing relative telomere length (rTL) in the Western‐Canaries Lizard (*Gallotia galloti*) across diverse environments over 10 years. Using mixed‐effect model‐averaging and hierarchical partitioning, we assessed the effects of intrinsic morphological (sex and body length) and extrinsic environmental (elevation, radiant sky temperature, wind speed and relative humidity) factors while controlling for temporal (year sampled) effects on rTL variation. In addition, we investigated temporal signals corresponding to extreme weather events over the sampling period. Intrinsic factors had the strongest influence, with males exhibiting shorter rTL than females, and females showing shorter rTL with increasing size. Temporal patterns revealed a negative correlation with dry years, indicating that even though environmental drivers may be secondary predictors compared to individual determinants, severe weather conditions may represent cumulative burdens. Multiple intrinsic and extrinsic variables, including climate, should be considered when investigating telomere dynamics in ectotherms.

## Graphical abstract

Telomere dynamics in ectotherms remain poorly understood relative to endotherms, yet are increasingly relevant in the context of climate change. Here, we used model‐averaged GLMMs and hierarchical partitioning to disentangle intrinsic and extrinsic drivers of relative telomere length in the Western‐Canaries Lizard (*Gallotia galloti*) across a 10‐year, multi‐locality dataset spanning Tenerife's elevational gradient. Intrinsic factors, particularly sex and body size, were the dominant predictors, while temporal analysis revealed a negative association between telomere length and drought years, highlighting the cumulative physiological burden of extreme weather events in wild ectotherm populations.

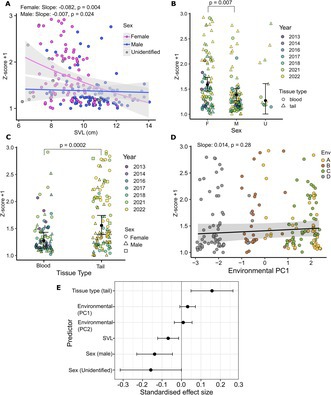

## 1 Introduction

Telomeres are protective caps at chromosome ends that reflect processes related to cellular ageing and organism longevity. While cellular division typically shortens telomeres due to incomplete replication (Olovnikov 1973), enzymes like telomerase can counteract this effect (Calado 2014). In endotherms, telomere shortening rate, rather than absolute length, better predicts longevity, depending on the chromosome (Whittemore et al. 2019; Karimian et al. 2024). Telomere length (TL) varies extensively across species, populations and individuals shaped by intrinsic factors such as ageing, sex, reproductive effort and resource availability (fat reserves), as well as extrinsic factors including environmental conditions (Monaghan et al. 2022; Tobler et al. 2022). In ectotherms like lizards, these dynamics are especially complex, with both shortening and lengthening observed in response to stress, food availability and reproductive demands (Axelsson et al. 2020; Olsson et al. 2018b, 2025). Understanding how such intrinsic and extrinsic drivers interact is central to linking telomere biology with broader questions of life‐history, evolution and physiological trade‐offs.

Several frameworks provide context for interpreting telomere dynamics. The pace‐of‐life hypothesis predicts that species with faster life histories experience accelerated telomere shortening due to higher metabolic demands (Giraudeau et al. 2019; Dantzer and Fletcher 2015; Friesen et al. 2022). Similarly, oxidative stress theory attributes telomere erosion to reactive oxygen species (ROS) accumulated during metabolism (Monaghan 2014; Ahmed and Lingner 2018; von Zglinicki 2002), particularly relevant in ectotherms, whose metabolism directly depends on external conditions (Ritchie and Friesen 2022; Burraco, Orizaola, et al. 2020; Friesen et al. 2022). These ideas integrate with life‐history theory, which connects telomere regulation to trade‐offs among growth, reproduction and survival, and highlights the roles of reproductive strategy, energy allocation and individual variation (Olsson et al. 2025; Monaghan 2010; Fitzpatrick et al. 2021). Heritability and selection add an evolutionary dimension to the study of telomere biology (Olsson et al. 2011a; Olsson et al. 2018b). These frameworks are relevant when investigating multiple drivers of telomere dynamics.

Unlike mammals, in which telomeres generally shorten with age, ectotherms exhibit more variable or even opposite patterns (Tobler et al. 2022; Monaghan et al. 2022; Mira‐Jover et al. 2024). In some lizards (*Psammodromus algirus*, *Lacerta agilis*), larger and older individuals have longer telomeres, potentially due to selective mortality or sustained telomerase expression (Burraco, Comas, et al. 2020; Axelsson et al. 2020), while others, like *Chlamydosaurus kingii* , show a curvilinear relationship with age (Ujvari et al. 2017). In the lizard *Gallotia galloti*, no correlation with age was found (Serén et al. 2023), aligning with that of some other ectotherms (Gao and Munch 2015; Lund et al. 2009). These mixed results complicate the relationship between telomere dynamics and life history strategies of reptiles, making a clear emergent hypothesis unclear (Morinha et al. 2020). Sexual dimorphism and reproductive strategy in lizards further modulate TL variation. Males are typically larger, with some exceptions (Braña 1996; Liang et al. 2022), but body size does not consistently predict TL. Females may maintain longer telomeres due to oestrogen‐mediated protection and lower competitive stress (Olsson et al. 2011a; Olsson et al. 2010; Bauch et al. 2020), potentially via the antioxidant effects of oestrogen and vitellogenin (Viña et al. 2005; Lindsay et al. 2020; Barrett and Richardson 2011). However, female TL remains sensitive to energetic costs of reproduction, including vitellogenesis and resource loss (through tail autotomy), which reduce the capacity for telomere elongation (Olsson et al. 2025; Hansson et al. 2024; Carretero 2007; Roig et al. 2000; Friesen et al. 2022). In males, by contrast, telomere elongation is linked to reproductive success, suggesting sex‐specific evolutionary trade‐offs, with a heritable basis (Olsson et al. 2011a; Olsson et al. 2025). While much of this evidence is based on cross‐sectional measures of relative telomere length (rTL), it provides mechanistic insight into observed sex differences in TL. Furthermore, TL also varies across tissues, with leukocytes typically exhibiting shorter telomeres than less proliferative tissues like muscle and skin, influencing ageing and disease susceptibility (Saferali et al. 2014; Chiu et al. 2016; Bhattacharya et al. 2019). However, in other studies, blood (including red blood cells and leukocytes) has been identified as a potential proxy in lizards, indicating a correlation with other tissue types (liver, heart, brain, spleen), despite absolute differences (Rollings et al. 2019).

Environmental conditions interact with these intrinsic drivers in shaping telomere dynamics. Elevation and associated climatic gradients affect ectotherm physiology (Anderson et al. 2022), but their influence on TL is inconsistent and context dependent. In some lizards, age structure explains more than environment (Burraco, Comas, et al. 2020), whereas in others, including *G. galloti*, longer telomeres at higher elevations suggest that cooler temperature may slow ageing and reduce oxidative stress (Serén et al. 2023; Fitzpatrick et al. 2021; Giraudeau et al. 2019). Conversely, elevated temperatures accelerate juvenile growth but shorten telomeres and induce oxidative damage, with transgenerational fitness costs (Zhang et al. 2023; Dupoué et al. 2022). Thermal and hydric microclimate thus appear as key regulators in shaping TL in wild populations, particularly under climate change (Maclean et al. 2021; Dupoué et al. 2020). Changes in elevation influence microclimate opportunities, and hence change thermoregulatory performance, having an effect on physiology, such as TL (Anderson et al. 2022). However, how this interacts with other intrinsic factors and fits into pace‐of‐life and life‐history frameworks remains unclear.

The Western‐Canaries Lizard (*Gallotia galloti*) provides an ideal model to assess intrinsic and extrinsic influences on TL. Native to Tenerife, it inhabits a heterogeneous landscape with elevational gradients and distinct bioclimatic zones (Algar and López‐Darias 2016; Albaladejo et al. 2022), ranging from arid coastal habitats to cooler, more humid regions in the north. The species occupies the widest elevational distribution of perhaps any lizard, from sea level to the peak of El Teide at 3718 m asl. consisting of three geographically separated subspecies, *G. g. insulanagae* (offshore islet), *G. g. galloti* and *G. g. eisentrauti*, which differ in their morphology and spatial distribution across the island, and hence contrasting environmental conditions.

*Gallotia galloti*'s ecological versatility and demonstrated links between oxidative stress, ecophysiology and adaptation (Gilbert, Žagar, et al. 2024; Serén et al. 2023, 2024; Gilbert, Megía‐Palma, et al. 2024) make it well suited for studying TL dynamics. All subspecies exhibit sexual size dimorphism, with males typically larger than females, and while largely wide‐foraging and herbivorous, polygynous mating behaviour characterised by male–male competition and territoriality has been observed (Molina‐Borja et al. 1997, 1998; Molina‐Borja and Rodriguez‐Dominguez 2004; Huyghe et al. 2005, 2010). These sex‐specific life‐history strategies are associated with differences in growth rates, reproductive investment and physiological stress (Meter et al. 2020; Steele and Warner 2020), all of which are associated with telomere dynamics. Prior research on male *G. galloti* suggests longer TL at higher elevations, with no correlation with age (Serén et al. 2023). Our study expands on this by incorporating both sexes, across multiple years and microclimate variables. Given Tenerife's spatial heterogeneity, we hypothesise that environmental factors will be the strongest predictors of TL, with longer TL at higher elevations, alongside sex and size, with bigger males having shorter TL, as seen in other lizards. We further predict that extreme hot and dry years will correspond to shorter telomeres in the same year due to increased physiological stress.

## 2 Materials & Methods

**FIGURE 1:**
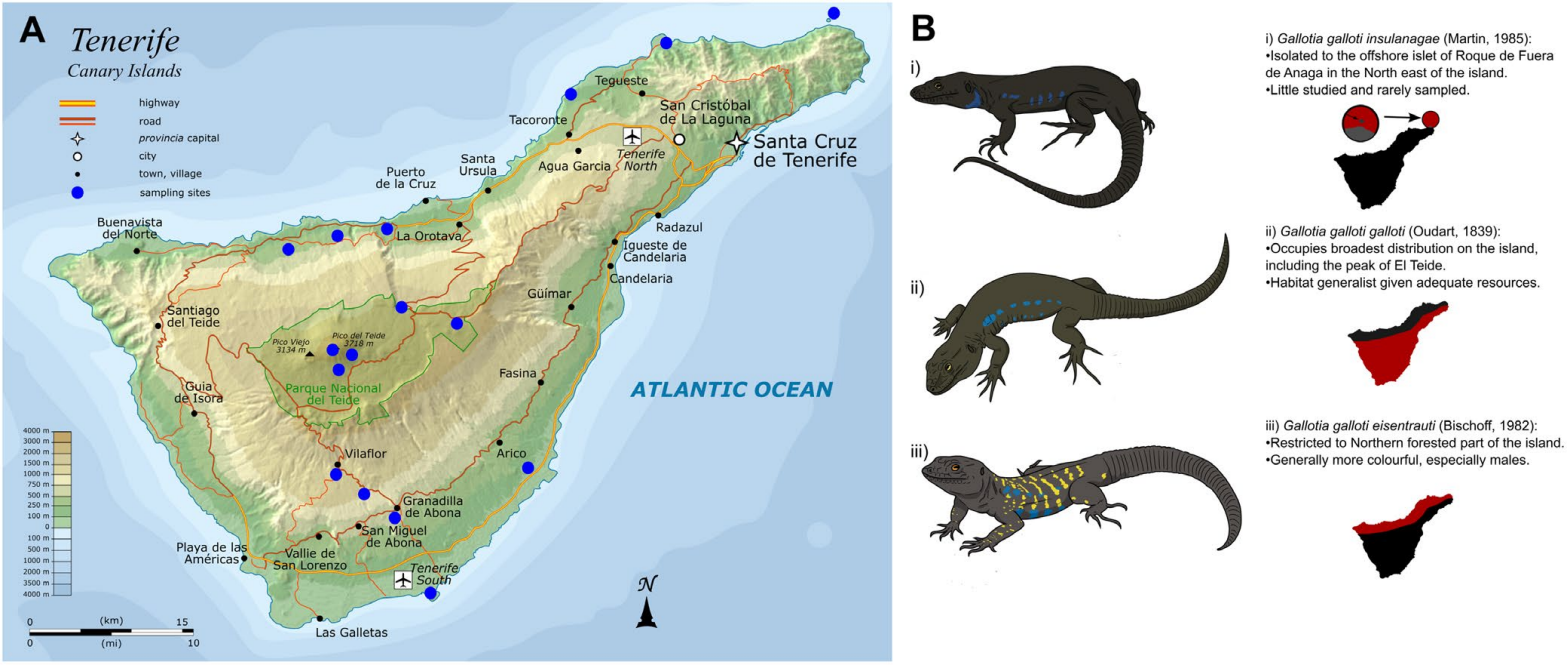
(A) Map of Tenerife including major landmarks and topography, with the summit of El Teide (3718 m asl) as the center of the island, and spatial sampling points marked with blue dots. Base map adapted from Oona Räisänen (Mysid—Wikipedia) 2010 under Creative Commons Attribution‐ShareAlike 3.0 Unported Licence. Modifications: Added sampling points. (B) Describes the three subspecies found on Tenerife including their approximate geographic distribution. Lizard images by Anna Maka, reproduced with permission.

Study sites span the elevation‐induced climate gradient on Tenerife, encompassing multiple localities across elevations and bioclimatic zones (Figure 1, Table S3). DNA was extracted from 236 samples (tail tips and dried blood spots) across 19 localities over 10 years, using a salt‐extraction method (Bruford et al. 1992); previously described in Gilbert, Megía‐Palma, et al. (2024) and Gilbert, Žagar, et al. (2024). Sample metadata containing locality, year, tissue type, sex and snout‐to‐vent length (SVL) is found in the associated Dryad data repository, and sample sizes across groups are described in Tables S2 and S3. Individuals of ambiguous sex were labelled “undetermined”. rTL was determined via quantitative polymerase chain reaction (qPCR), calculating the telomere repeat copy number to single copy gene (18S rRNA, abbreviated as SCG) number ratio (Cawthon 2002). Standardised DNA elutions were combined with SYBR Select Master Mix (Applied Biosystems), ddH_2_O and SCG/telomere primers (Supplementary Methods). Quantification was performed in triplicate on a ThermoFisher QuantStudio Flex qPCR (conditions in Supplementary Methods), using a pooled “Gold standard” DNA sample and a no‐template control in all runs for standardisation (Cawthon 2002).

LinRegPCR (Ramakers et al. 2003) corrected amplification curves with constant fluorescence thresholds kept across all plates (0.171 in telomere plates, 0.144 for SCG plates).

The Pfaffl equation was used to calculate rTL, normalising transcripts from telomere regions to housekeeping (SCG) regions (Pfaffl 2001) (Supplementary Methods). To facilitate comparability across studies, *Z*‐scores were calculated by mean‐centering raw rTL values by subtracting the overall mean and subsequently scaling by the standard deviation (Verhulst 2020). This standardisation yields values with a mean of zero and a standard deviation of one, expressing variation in rTL in units of standard deviation. Adherence to MIQE 2.0 guidelines (Bustin et al. 2025), where appropriate, is demonstrated in Table S1.

### 2.1 Microclimate Variables

Microclimatic parameters were modelled using the *microclima* (Maclean et al. 2019) and *NicheMapR* (Kearney et al. 2021) packages in R for each locality (< 5 m resolution) for each year, following (Gilbert, Žagar, et al. 2024). The model used is predictive but was originally ground‐truthed with dataloggers (Maclean et al. 2019), however has not been validated at the study site. Monthly values were aggregated into mean, maximum and minimum annual values for each locality. Correlated environmental variables were summarised using principal component analysis (PCA) implemented with the *prcomp* function in base R. Environmental variables were centred and scaled prior to PCA, and leading principal components explaining the majority of variance were retained for subsequent analysis (Tables S4 and S5).

### 2.2 Statistical Analyses

Telomere length was analysed using generalised linear mixed‐effects models (GLMMs) fitted with the glmmTMB package (McGillycuddy et al. 2025). Models were fitted with a Gamma distribution and log‐link function, requiring a transformation (+1) to ensure positive values. Samples with missing data were removed. To account for temporal non‐independence among samples, year was included as a random intercept. Fixed effects included the first two environmental principal components (85% variance explained), SVL, sex, tissue type and subspecies (also referred to morphotype). A biologically motivated interaction between SVL and sex was included to allow for sex‐specific scaling relationships.

A global model containing all candidate predictors was subjected to automated model dredging using the *dredge* function in the MuMIn package (Bartoń 2025). Models were ranked by Akaike's Information criterion corrected for small sample size (AICc). Inference was based on model‐averaged coefficients calculated across the supported model set using *model.avg*, thereby accounting for model selection uncertainty, which supports recovery of degrees of freedom regarding overparameterization in evolutionary ecology studies (Hegyi and Garamszegi 2011).

Fixed effects were derived from model‐averaged estimates on the link scale, while predicted responses were generated on the response scale for interpretation and visualisation. Estimated marginal means and continuous marginal trends were obtained using the *emmeans* package (Lenth 2024), allowing the calculation of predicted TL across observed gradients of continuous predictors and among categorical factor levels. Confidence intervals were calculated using asymptotic standard errors and back‐transformed where appropriate.

Effect sizes for continuous predictors were interpreted directly from model‐averaged slopes, while categorical predictors (sex, tissue type, morphotype) were interpreted via contrasts relative to reference levels.

An alternative model selection framework based on gamma‐distributed GLMs with exhaustive model selection across predictors (including year as a fixed effect) was also used to identify key correlates of TL, with full details and results provided in the Supporting Informations.

Hierarchical model partitioning using the *hier.part* function from the *hier.part* R package (Nally and Walsh 2004) quantified independent predictor effects, followed by a randomisation test to assess the statistical significance of each independent contribution. To compare intrinsic (independent‐level variables: sex and SVL) and extrinsic (group‐level environmental principal components) predictors, Nagelkerke's *R* ^2^ and deviance explained for respective partial models were calculated.

Sensitivity analyses using subsets of data were used to validate the main inferences due to inclusion of multiple variables, some of which had small sample sizes. This included post hoc results with unidentified sexes removed, locality as a random effect, model subsets of tissue types (and year x tissue type interactions), and model subsets of morphotypes (including with *G. g. insulanagae* removed).

Heatwave and drought frequencies from AEMET (Agencia Estatal de Meteorologia) weather stations were calculated as the top 10% of maximum and mean temperature per month, and bottom 10% of total precipitation per month as in Gilbert, Megía‐Palma, et al. (2024) and Gilbert, Žagar, et al. (2024). Cross‐correlation coefficient analysis (*ccf* function in base R) was used to determine the lag between the weather events and rTL, with a maximum lag of 6 years and 5000 permutations for significance testing.

## 3 Results

Relative TL varied across sampling localities but showed considerable overlap, suggesting additional influencing factors were likely to play a dominant role (Figure S1). Environmental variables were reduced to 5 principal components (Tables S4 and S5), where the first two representing 86% of environmental variation were used in modelling. Principal component 1 (66% of variance) represents a broad elevational‐microclimatic gradient, with strong contributions from elevation and relative humidity (both ~27%), and opposing contributions from radiant sky temperature (~28%) and windspeed (~15%), while principal component 2 (19% of variance) is dominated almost entirely by solar radiation (~94%) (Tables S4 and S5).

Variation in TL was analysed using Gamma‐distributed GLMMs with a log link, including year as a random intercept. Model selection based on AICc identified three competing models within △AICc < 2, together accounting for 99% of the cumulative model weight (Table S6). Across these models, sex, SVL and tissue type were consistently retained, whereas environmental predictors and morphotype received comparatively weaker support.

The results were mirrored in the alternative model selection framework through GLMs with exhaustive model selection across predictors (including year as a fixed effect instead of a random effect); however, year was included in the final model (Table S8).

Model‐averaged estimates revealed significant effects of sex, body size and tissue type on TL (Table 1). TL decreased with increasing SVL, indicating shorter telomeres in larger individuals. Males exhibited significantly shorter telomeres than females, representing the strongest effect among the predictors. Tissue type significantly influences TL, with tail tissue samples exhibiting longer TL than dried blood samples.

**TABLE 1:** Final model estimates and significance showing each model variable, estimate value, standard error, *t*‐value, *p*‐value and significance.

Predictor	Estimate	Adjusted SE	*z*	*p*
Intercept	0.851	0.170	4.99	< 0.001
Env_PC1	0.007	0.011	0.65	0.516
Sex (male)	−0.131	0.046	−2.82	0.0048
Sex (unknown)	−0.163	0.080	−2.04	0.0418
SVL	−0.046	0.017	−2.74	0.0062
Tissue type (tail)	0.140	0.055	2.56	0.010
Morphotype (*galloti*)	−0.009	0.029	−0.31	0.757

Environmental effects were weak. The first environmental principal component (Env_PC1) showed a positive but non‐significant association with TL under both full and conditional model averaging, and morphotype did not have a detectable effect. Pairwise comparisons based on marginal means further supported sex and tissue‐specific differences, while indicating no strong evidence for interaction‐dependent shifts across the observed SVL range.

**FIGURE 2:**
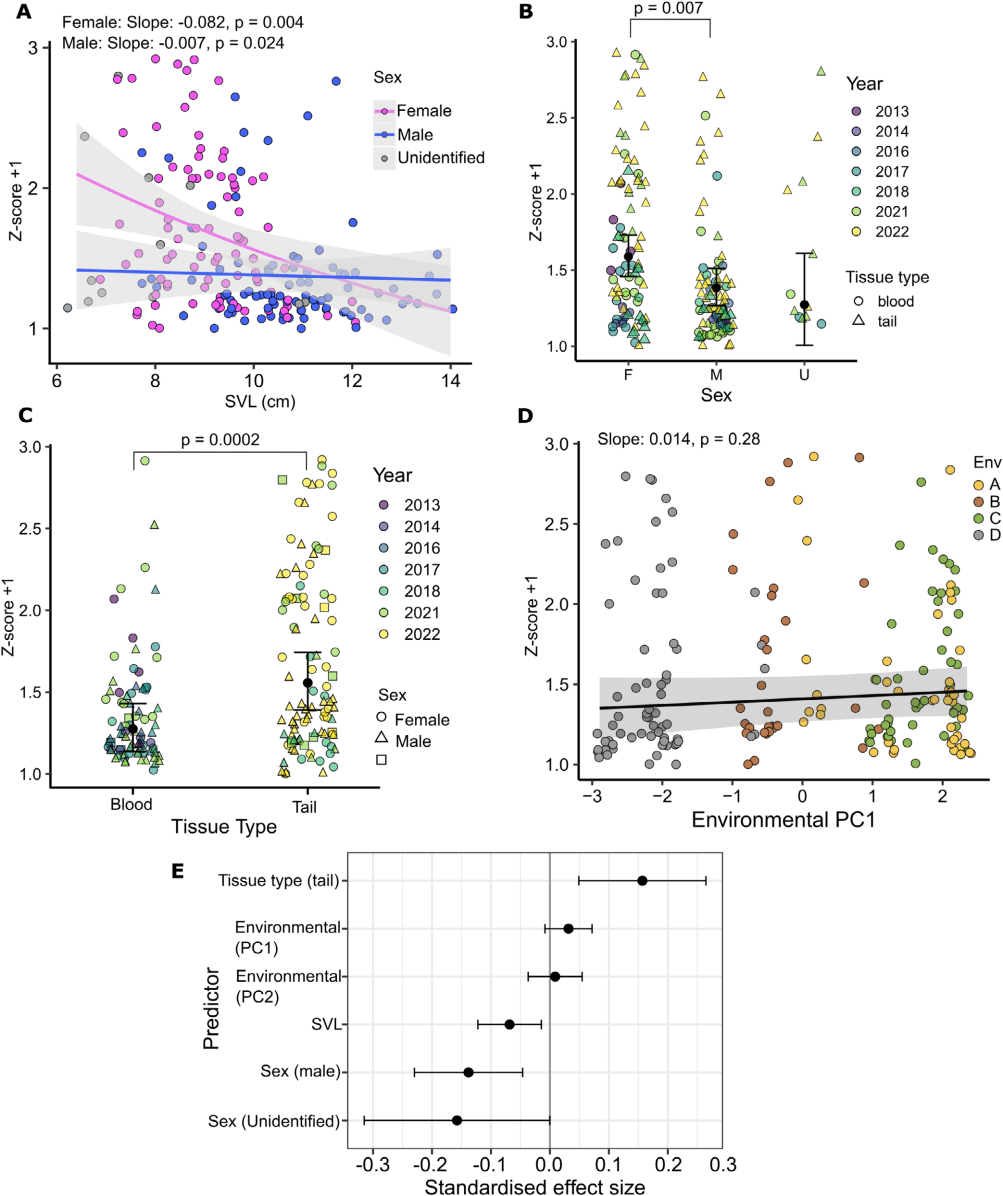
Relative telomere length transformed to positive *z*‐score against variables included in the final model. Points are coloured by sex in (A), year in (B and C), and environment type in (D). Coefficients and significance for continuous variables (SVL and Env PC1) are taken from the model with 95% confidence intervals, and are GLMM‐predicted means on the response scale. The reported slope and *p*‐values are fixed effect estimates from the gamma GLMM (log link). Factor variables are tested for significance with estimated marginal means, and Tukey adjusted *p*‐values are shown where significant. Mean points are shown as black points on 2B, 2C and standard error on (B and C). (E) Coefficients and standard error for each predictor standardised to calculate effect size on the log (link) scale, with distance from zero representing magnitude of effect, and positive/negative representing direction of correlation.

Relationships between TL (shifted *z*‐score) and predictors retained in the supported mixed‐effects models are illustrated in Figure 2. TL declined with increasing SVL across both sexes, with a steeper negative slope in females (slope = −0.082, *p* = 0.004) compared to a significant but biologically irrelevant rate of change in males (slope = −0.007, *p* = 0.024; Figure 2A). Consistent with this pattern, males exhibited significantly shorter telomeres than females when averaged across body size (Tukey‐adjusted *p* = 0.009; Figure 2B), while individuals of unidentified sex did not differ significantly from either group. Tissue type had a marked effect on TL, with tail tissue samples exhibiting higher TL than blood samples (Tukey‐adjusted *p* = 0.0002; Figure 2C). In contrast, the first environmental principal component (Env_PC1, containing high factor loadings for elevation [−0.52], relative humidity [−0.52] and radiant sky temperature [0.53]) did not correlate with TL (slope = 0.014, *p* = 0.28; Figure 2D). Standardised effect sizes derived from the model‐averaged estimates indicated that sex and tissue type had the largest effects on TL, whereas SVL showed a moderate negative effect and environmental principal components had small and imprecise effects centred near zero (Figure 2E).

Hierarchical partitioning revealed tissue type (33.5%) and SVL (31.7%) were the most influential predictors, shortly followed by sex (28.3%) (Figure S2). Both environmental principal components contributed negligible amounts (4.1% and 2.3% respectively). Randomisation tests indicated all predictors' independent contributions were significant except for the two environmental principal components (Table S9). Model fit metrics confirmed intrinsic variables (sex, SVL) explained more variance than extrinsic variables (environmental PCs), highlighting greater explanatory power in the context of the full model (Table S7).

**FIGURE 3:**
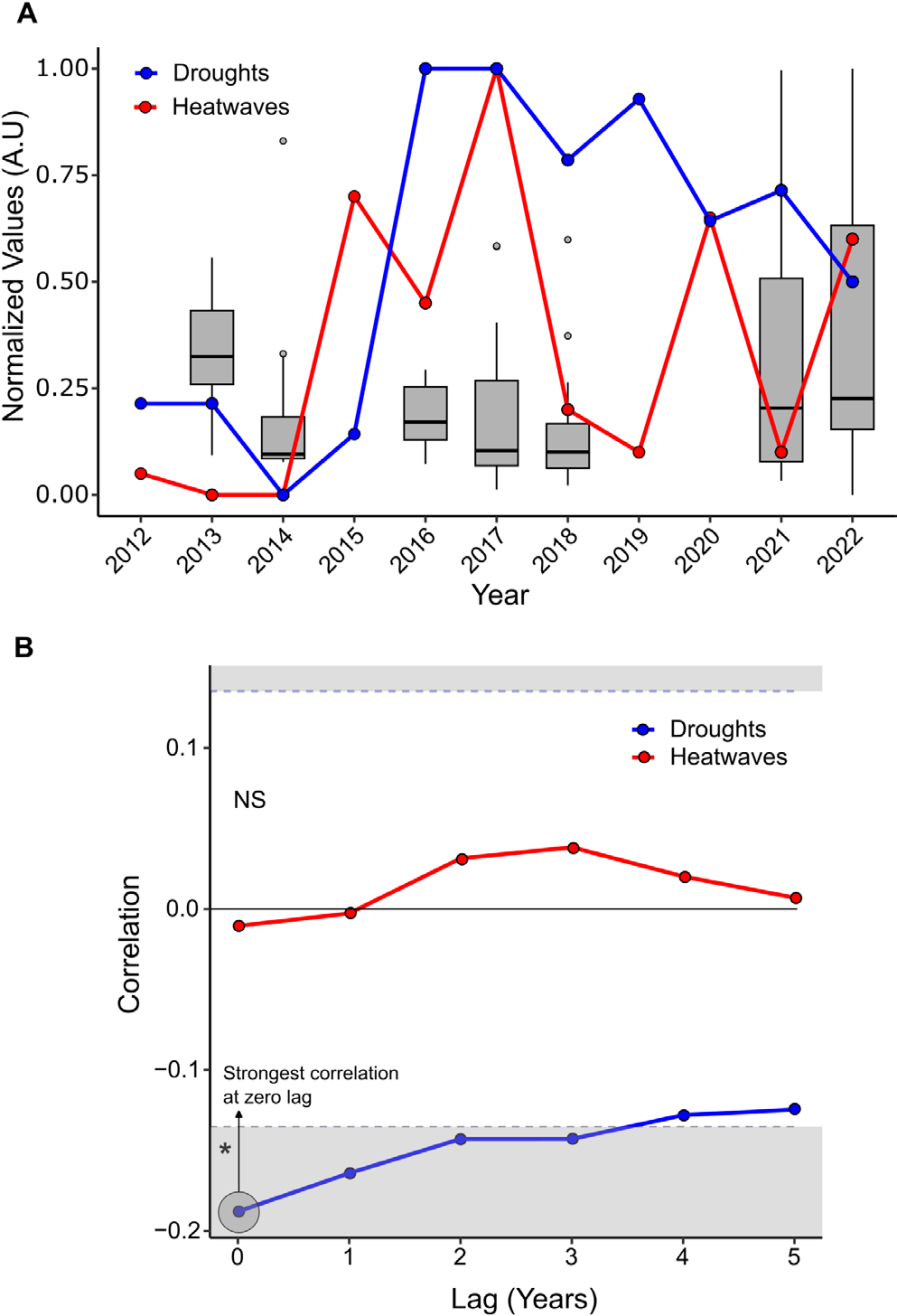
Cross correlation of climate events and *z*‐score (rTL). (A) *z*‐scores, heatwave count and drought count, scaled via min‐max normalisation for each year (arbitrary units). The *y*‐axis therefore represents the *z*‐score and the number of extreme weather events along an arbitrary scale. (B) Cross‐correlation analysis between *z*‐score and extreme climate events. The *Y*‐axis indicates the degree of correlation, and the *X*‐axis indicates the amount of lag years. Dashed lines indicate significance thresholds, with greyed areas representing the area of statistical significance. The asterisk * indicates statistical significance verified with permutation testing (drought, *p* = 0.004, heatwaves, *p* = 0.8778). At zero‐lag the correlation is strongest, meaning the years with more droughts are the same years with the shortest telomeres, and vice versa.

To assess the relationship between collection year and rTL, cross‐correlation analysis examined the impacts of dry and hot periods occurring in these years. Relative TL was shortest in the driest years (zero lag), with elevated dry periods from 2016 to 2019 (Figure 3A) negatively correlated with rTL and weakening over time (Figure 3B). This crossed the significance threshold on the cross‐correlation analysis, verified through permutation testing (5000 permutations, *p* = 0.004). No significant relationship was detected between extreme heat events and rTL (5000 permutations, *p* = 0.8778).

## 4 Discussion

The multivariate interactions of TL in ectotherms provide insight into their biology, ecology and response to environmental change. Because TL is associated with somatic maintenance, oxidative stress and survival probability, shorter telomeres are generally interpreted as reflecting increased physiological wear, whereas longer telomeres may indicate greater investment in maintenance and potential longevity. Telomere shortening and lengthening occur due to developmental, environmental and heritable factors. The consequences of this vary by ecological context and life history. Here, we investigated drivers of TL in a spatial–temporal dataset of *G. galloti* on Tenerife using mixed effect model‐averaging and hierarchical partitioning, assessing morphological characters, elevation‐induced environmental effects and influence of drought and heatwave years.

### 4.1 Sex, SVL and Tissue Type as Intrinsic Drivers

Models identified sex and SVL as significant intrinsic drivers of rTL variability. Despite the rejection of an interaction term between these variables from model selection, the autocorrelation of these factors still makes it difficult to identify the weight of each variable explaining rTL. Females generally had longer telomeres, a trend reflected in some reptiles due to genetic and hormonal factors such as telomeric repeats on the W chromosome (Singchat et al. 2018, 2019; Matsubara et al. 2015) and female‐specific hormones (Singchat et al. 2019; Ballen et al. 2012; Olsson et al. 2011a; Ensminger et al. 2021). Conversely, male breeding behaviour can lead to higher corticosterone levels (Olsson et al. 2010), resulting in sex‐specific stress‐adjacent evolutionary strategies associated with reproductive success (Olsson et al. 2011b). Sex also interacts with size, where different size classes show varying trajectories of TL (Axelsson et al. 2020; Olsson et al. 2010), also reflected in our data. All individuals were adults, with SVL serving as a proxy for relative age such that larger individuals represent older age cohorts, based on established SVL‐age relationships in *G. galloti* (Gilbert, Megía‐Palma, et al. 2024). This relationship is based on an equation between age structure and SVL determined in prior literature; however, it may be inaccurate (Castanet and Báez 1991; Serén et al. 2023).

Larger ectotherms may have longer telomeres (Rollings et al. 2020; Burraco, Comas, et al. 2020; Ujvari et al. 2017; Axelsson et al. 2020), contrary to trends in endotherms (Ringsby et al. 2015; Scott et al. 2006; Pepke and Eisenberg 2022; Foote et al. 2011; Beaulieu et al. 2017), and telomeres are dynamic through oxidative erosion and telomerase activity (Fitzpatrick et al. 2021). Our results contradict expectations that larger lizards (particularly females) have shorter telomeres, whose subjacent ecological reasons require further study.

Tissue type showed tail tips having longer telomeres than blood, potentially due to differences in cellular turnover and localised oxidative stress (Daniali et al. 2013). The different storage conditions of these tissues may also play a role; therefore, biological explanations should be cautiously inferred. In Egyptian fruit bats ( *Rousettus aegyptiacus* ), wing skin TL correlated with tissues but not blood, making it a reliable non‐lethal proxy (Power et al. 2021). Similarly, reptile tail tips may serve as proxies, though comparative data are limited. Regenerated tail tissue may have longer TL due to post‐autotomy telomerase activation (Pacoń et al. 2024; Fitzpatrick et al. 2019), though tail loss was not recorded in this study. The inclusion of tissue type bears less physiological or ecological importance, but describes an important methodological consideration during sample collection, particularly in studies collating data across sampling expeditions. These sex and size‐dependent differences suggest that telomere dynamics reflect divergent life‐history strategies, where females may incur greater physiological costs associated with reproduction, while larger (older) individuals do not seem to accumulate telomere attrition consistent with ageing‐related somatic decline, perhaps due to mechanisms associated with environmental (and hence physiological) tolerance breadth (Gilbert, Megía‐Palma, et al. 2024; Gilbert, Žagar, et al. 2024; Serén et al. 2023, 2024).

### 4.2 Environmental and Temporal Effects

Here, we distinguish between microclimate (fine‐scale spatial variation across elevations) and macroclimate (temporal variation including extreme drought or heatwaves) to clarify their potentially different effects on TL. At the spatial scale, microclimate had minimal explanatory power. Environmental influences on TL are known (Fitzpatrick et al. 2021; Serén et al. 2023; Axelsson et al. 2020) however, here they were reduced when including sex and SVL, suggesting *G. galloti* effectively buffers oxidative state across elevations on Tenerife, potentially limiting oxidative‐induced telomere erosion and hence a lesser effect of environment (Gilbert, Megía‐Palma, et al. 2024; Gilbert, Žagar, et al. 2024). Buffering from harsh environments is therefore very dependent on the sex and size of the lizard, with these variables being more important than external conditions in this study (Megía‐Palma et al. 2020). If *G. galloti* is well‐adapted to diverse environments, it will be more resilient to extreme climate events and respond rapidly, with TL being a potential indicator of this (Gilbert, Megía‐Palma, et al. 2024).

Temporal variation explained more TL variance than spatial effects when included as a fixed effect. Further, when correlated with extreme weather events, dry conditions showed a negative effect on rTL, with the driest years (2016–2018) yielding shorter TL than wetter, cooler years (2021–2022). Extreme temperatures and drought accelerate telomere attrition (Seeker et al. 2021; Chatelain et al. 2020), likely pivoting on stress‐related physiology (Chatelain et al. 2020; Angelier et al. 2018). Drought may also indirectly affect TL through food availability and habitat quality, which are often compromised during drought periods (Angelier et al. 2018; Wilbourn et al. 2017; Olsson et al. 2025). Water availability appeared more critical than heat, implying better adaptation to thermal rather than hydric stressors (Dupoué et al. 2020). This highlights that single‐time‐point, site‐level microclimate measures may underestimate the impact of environmental stress on telomere dynamics, whereas longitudinal macroclimate assessment captures more physiologically meaningful variation with potential consequences for survival and reproductive output.

Besides immediate effects of dry conditions on shortening telomeres, there could also be long‐term developmental and evolutionary effects. Thermal conditions during early life shape TL long‐term (Olsson et al. 2018a), possibly contributing to observed longer rTL in 2021 and 2022 (Ton et al. 2023). Climate events with strong droughts around 2017 may have affected *G. galloti*'s genetic composition in 2022 (~1 generation), inducing oxidative stress during egg and early life stages (Gilbert, Megía‐Palma, et al. 2024). This possibly selected against individuals with shorter telomeres (Fitzpatrick et al. 2021; Asghar et al. 2015), and better ROS mitigation prevented telomere attrition throughout 2017–2022. This aligns with suggestions that shorter TL in declining lizard populations may be inherited or influenced by the environment at vulnerable embryonic stages (Dupoué et al. 2022). The study that showed shorter TL in declining lizard populations was done in a viviparous species that is very sensitive to dehydration (Dupoué et al. 2022), while *G. galloti* is an oviparous species with better abilities to resist dry conditions (Vernet et al. 1995), and likely better copes with increasing drought pressures in the environment as suggested by our results. It should be noted that our analysis of temporal variation only provides correlational evidence and may be influenced by other factors such as sample size or variables included in the model. In addition, molecular markers including rTL may not necessarily be indicative of realised fitness or survival, and should be considered with other lines of evidence for more robust assertions about telomeres' role in population ecology. This kind of longitudinal environmentally driven telomere shortening may have downstream consequences for survival and future reproductive output, particularly if repeated exposure to drought accelerates cumulative physiological stress within cohorts. This effect may be masked by single‐time point microclimate effects as in our main model and should be considered in future studies.

### 4.3 Individual Versus Population Level Effects

Hierarchical partitioning suggested that individual predictors (sex, SVL) influenced rTL more than population‐level differences at the environmental scale. This is unexpected given Tenerife's elevation‐driven climate gradient, which impacts some other biological aspects such as oxidative stress, protein expression and evolution in *G. galloti* (Gilbert, Žagar, et al. 2024; Serén et al. 2023; Gilbert, Megía‐Palma, et al. 2024), but on the other hand is in line with no variation found in thermal physiological performance (Serén et al. 2023). Previous studies found elevation‐related TL differences (longer at higher elevations) (Serén et al. 2023), however, our larger dataset (*n* ~ 200, across, but controlling for years) suggests individual‐level effects are stronger. Population‐scale TL differences occur across taxa, reflecting genetic and environmental variation (Kärkkäinen et al. 2022; Ly et al. 2019), nevertheless, individual‐level dynamics related to life‐history traits like reproductive investment, self‐maintenance and response to stressors differ even among genetically similar individuals (Fitzpatrick et al. 2021; Bakaysa et al. 2007; Romano et al. 2013). These cumulative factors illustrate how individual experiences can drive telomere dynamics (Boonekamp et al. 2014). If TL captures cumulative individual condition, the dominance of intrinsic predictors suggests that population‐level responses to environmental change may emerge indirectly through shifts in demographic structure (e.g., sex ratios, age structure), rather than through uniform physiological responses across individuals, especially when considering a species like *G. galloti* that occurs across various environmental conditions.

## 5 Conclusion

Our findings highlight the importance of multivariate approaches in understanding telomere dynamics in ectotherms like *G. galloti*, where physiological and environmental interactions are complex. Despite environmental predictors playing a minor role in TL across the landscape, their indirect or temporal effects remain relevant. Future research should incorporate longitudinal data to track individual telomere trajectories and further disentangle intrinsic (genetics, morphology) and extrinsic (climate, resource availability) influences.

This research demonstrated intrinsic factors were the strongest predictors, likely reflecting sex‐specific hormones, life‐history strategies or genetics. The temporal trend correlated with dry years, suggesting environmental factors still shape TL at broader scales. Longer term sampling designs to assess the connection between climate variability (particularly extreme weather events) and TL should investigate this relationship further. Lastly, lacertids like *G. galloti*, with their broad elevational ranges, offer valuable systems for studying molecular markers of population health, such as TL.

## Author Contributions

**Edward Gilbert:** conceptualization (equal), data curation (equal), formal analysis (equal), investigation (equal), validation (equal), visualization (equal), writing – original draft (equal), writing – review and editing (equal). **Megan L. Power:** data curation (equal), formal analysis (equal), investigation (equal), methodology (equal), writing – review and editing (equal). **Annika Wolberg:** data curation (equal), formal analysis (equal), investigation (equal), writing – review and editing (equal). **Rodrigo Megía‐Palma:** resources (equal), writing – review and editing (equal). **Anamarija Žagar:** resources (equal), writing – review and editing (equal). **Marta López‐Darias:** resources (equal), writing – review and editing (equal). **Miguel A. Carretero:** resources (equal), writing – review and editing (equal). **Nina Serén:** resources (equal), writing – review and editing (equal). **Pedro Beltran‐Alvarez:** conceptualization (equal), data curation (equal), formal analysis (equal), investigation (equal), supervision (equal), validation (equal), visualization (equal), writing – original draft (equal), writing – review and editing (equal). **Katharina C. Wollenberg Valero:** conceptualization (equal), data curation (equal), formal analysis (equal), investigation (equal), methodology (equal), supervision (equal), validation (equal), visualization (equal), writing – original draft (equal), writing – review and editing (equal).

## Funding

E.G., P.B.‐A. & K.C.W.V. were supported by the Leeds‐York‐Hull Natural Environment Research Council (UKRI/NERC) Doctoral Training Partnership (DTP) PANORAMA under grant NE/S007458/1. AŽ was funded by the Slovenian Research and Innovation Agency (ARIS) (P1–0255 and MN‐0004‐105). M.A.C. acknowledges projects of MINECO (Spain) /ERDF CGL2015‐67789‐C2‐1‐P and PGC2018‐097426‐B‐C21, FCT (Portugal)/ERDF 28014 02/SAICT/2017 and 2022.03361.PTDC. M.L.P. & K.C.W.V. acknowledge funding by the European Union (ERC, MolStressH2O, #101044202). Views and opinions expressed are however those of the author(s) only and do not necessarily reflect those of the European Union or the European Research Council Executive Agency. Neither the European Union nor the granting authority can be held responsible for them. A.W. received funding from Arcadia University (USA) to perform lab work in UCD.

## Ethics Statement

Ethical approval of research for EG and MLD was obtained from the University of Hull, FEC_2022_103. The Spanish Dirección de Agricultura, Ganadería y Alimentación of Consejería de Medio Ambiente y Ordenación del Territorio of Comunidad de Madrid provided ethical approval (PROEX 128/19) after clearance of the Ethical Committee of CSIC and approval of Área de Protección Animal of Comunidad de Madrid. CAM provided a certificate for animal experimentation to RMP (0945555854754694309162), and Generalitat de Catalunya provided a certificate for animal experimentation to MAC (27/07/2001). EG and MLD collection permits were obtained from Cabildo de Tenerife Sigma 1701‐22 AFF 154‐22. Gobierno de Canarias and Cabildo Insular de Tenerife provided specific permits AFF469/13 (2013‐02234), AFF 51/16 (2016‐00480) to study lizards to capture *G. galloti* across the island. This included specially protected areas such as Teide National Park and the Integral Natural Reserve of Roques de Anaga: 369/2014 (2014/2721), AFF 218/14 (2014–01172/29679), MDV/amp, AFF 110/162016–01215, JLRG/arm, no. 3/332, YMG/cpa, AFF 57/172017–00308, AFF 67/172017–00335, JLRG/arm (138/2). Collection permits for samples of 2021 were obtained from Cabildo de Tenerife Sigma 2021–01359 AFF 146–21 and Gobierno de Canarias Ref. Expte. 2021/26145, and for 2018 samples from Cabildo de Tenerife Sigma 2018‐02258 AFF 160‐18. Sampling permits AFF 57/172017‐00308, exit record: 13413, AFF 67/172017‐00335, exit record: 15807, AFF 160/182018‐02258, exit record: 28466, AFF 146/21 (2021‐01359) and 2021/26145; and Teide National Park sampling permit JLRG/arm, exit record 138/2 issued by Cabildo Insular de Tenerife.

## Conflicts of Interest

The authors declare no conflicts of interest.

## Supporting information


**Table S1:** Checklist for MIQE guidelines (Bustin et al. 2025) for transparency of qPCR data.
**Table S2:** Sample sizes for each variable used in analyses.
**Table S3:** Sample sizes for each locality sampled (across years).
**Table S4:** Variance explained and cumulative variance for each principal component.
**Table S5:** Raw loading contribution of each environmental variable, and calculated percentage contribution.
**Table S6:** Top three models. Models are ranked by AICc; all include a random intercept for year.
**Table S7:** Model fit of pseudo *R* ^2^ (Nagelkerke) and deviance explained for the full model, a model with just the intrinsic variables, and just the external variables
**Table S8:** Alternative generalised linear model including year as a fixed effect and all environmental variables after model selection. Shown are final model variables, estimates, standard error, *t*‐value, *p*‐value and significance.
**Table S9:** Randomisation test of hierarchical partitioning examining statistical significance of the independent contributions of each predictor variable to the log‐likelihood goodness‐of‐fit.
**Figure S1:** Relative telomere length (transformed to *z*‐scores) across different localities. Raw data, where each point is a single animal, is coloured by the environment type. Roque de Anaga (RA) is considered separately as an islet in the NE, occupied by the understudied *G. g. insulanagae* subspecies.
**Figure S2:** Percentage of independent effects of predictors included in model on rTL, calculated using hierarchical partitioning. Independent effects are expressed as a percentage of the total explained variance.

## Data Availability

Data supporting this study are archived in a Dryad repository https://doi.org/10.5061/dryad.4f4qrfjt7. Data used and the full R scripts can also be found at: https://github.com/RepTed/Telomere‐dynamics‐in‐ggalloti.
