# Supplementary material for: Telomere Length Is Determined by Intrinsic Factors and Is Shortened During Drought Years in Gallotia galloti
Source: Ecol Evol. 2026 May 5;16(5):e73549. doi: 10.1002/ece3.73549 (PMC13139767; doi:10.1002/ece3.73549)
Supplement: Supplementary file 1 — Table S1: Checklist for MIQE guidelines (Bustin et al. 2025) for transparency of qPCR data. Table S2: Sample sizes for each variable used in analyses. Table S3: Sample sizes for each locality sampled (across years). Table S4: Variance explained and cumulative variance for each principal component. Table S5: Raw loading contribution of each environmental variable, and calculated percentage contribution. Table S6: Top three models. Models are ranked by AICc; all include a random intercept for year. Table S7: Model fit of pseudo R 2 (Nagelkerke) and deviance explained for the full model, a model with just the intrinsic variables, and just the external variables Table S8: Alternative generalised linear model including year as a fixed effect and all environmental variables after model selection. Shown are final model variables, estimates, standard error, t‐value, p‐value and significance. Table S9: Randomisation test of hierarchical partitioning examining statistical significance of the independent contributions of each predictor variable to the log‐likelihood goodness‐of‐fit. Figure S1: Relative telomere length (transformed to z‐scores) across different localities. Raw data, where each point is a single animal, is coloured by the environment type. Roque de Anaga (RA) is considered separately as an islet in the NE, occupied by the understudied G. g. insulanagae subspecies. Figure S2: Percentage of independent effects of predictors included in model on rTL, calculated using hierarchical partitioning. Independent effects are expressed as a percentage of the total explained variance. [file ECE3-16-e73549-s001.docx]

**Supplementary Materials to the manuscript “Telomere length is determined by intrinsic factors and is shortened during drought years in *Gallotia galloti*”**

Edward Gilbert^1,2^, Megan L. Power^3^, Annika Wolberg^3,4^, Rodrigo Megía-Palma^5^, Anamarija Žagar^6,7,8,9^, Marta López-Darias^10^, Miguel A. Carretero^6,7,11^, Nina Serén^6,7^, Pedro Beltran-Alvarez^12^, Katharina C. Wollenberg Valero^3,13*^

Author affiliations are references in the main text.

**Supplementary methods**

Sampling comprised 236 adult individuals of both sexes across 19 localities between 2013 and 2022, with repeated multi-year sampling conducted at eight localities, enabling assessment of both spatial variation and temporal telomere changes. Sampling took place between May and September within the active period of the lizard. Dried blood spots were stored at -20C on Whatmann FTA cards (2013-2021), and tail tip tissue (2016-2022) was stored at -20C in 99% ethanol. Different tissue types were not sampled from the same lizard. A table detailing the sampling regime of which localities and which years, and from which tissue types is found in the Electronic Supplementary Materials via GitHub.

**Lab**

DNA elutions were standardised to 20 ng/μl, and 2 μl was combined with 10 μl SYBR Select Master Mix (Applied Biosystems), 4.9 μl ddH_2_O, with 1.55 μl forward primer and 1.55 μl reverse primer targeting telomeric repeats (Tel1: 5′-CGGTTTGTTTGGGTTTGGGTTTGGGTTTGGGTTTGGGTT-3′ and Tel2: 5′-GGCTTGCCTTACCCTTACCCTTACCCTTACCCTTACCCT-3′, which have previously worked on *G. galloti*: [(Serén et al. 2023)](https://paperpile.com/c/51ncpv/bCToQ) for both the SCG reactions and telomere reactions. For the reference SCG, mitochondrial *18S* was selected with the following primers previously working on *G. galloti*: 18SF (5′-AAACGGCTACCACATCCAAG-3′) and 18SR (5′-CTCGATCCCAAGATCCAACT-3′) [(Serén et al. 2023)](https://paperpile.com/c/51ncpv/bCToQ). qPCR was performed on a ThermoFisher QuantStudio Flex qPCR system using the following protocol: 95 °C for 3 minutes, followed by 40 cycles of 95 °C for 30 seconds, 59 °C for 30 seconds, 72 °C for 30 seconds, followed by a melt curve.

Mean plate efficiencies quantified from LinRegPCR were 84.3% for SCG plates and 88.7% for telomere plates.

**Pfaffl equation**

$rTL = \frac{E{}_{TEL}^{CT tel (G.S) - CT tel (n)}}{E{}_{SCG}^{CT SCG (G.S)-CT SCG (n)}}$

Where: *E_tel_* and *E_SCG_* are the plate reaction efficiencies for the respective group calculated by LinRegPCR, *CT tel (G.S)* and *CT SCG (G.S)* are the mean gold standard CT values for telomeres and single copy genes, and *n* is the sample CT value.

Adherence to the MIQE guidelines, where appropriate, are demonstrated in Supplementary Table 1.

**Supplementary Table 1.** Checklist for MIQE guidelines [(Bustin et al. 2025)](https://paperpile.com/c/51ncpv/TJhe) for transparency of qPCR data.

| **MIQE Item** | **Included in Manuscript** |
| --- | --- |
| **Sample details** | Species, tissue types (tail tips, dried blood spots), n = 236, 14 samples removed, locality & year metadata (Electronic Supplementary) |
| **DNA extraction method** | Salt-extraction protocol cited (Bruford et al. 1992) |
| **Primer sequences** | Telomere and SCG (18S) primers provided in Supplementary Methods |
| **Primer validation** | The melt curves produced for both telomere and 18S after amplification by qPCR displayed a single peak, indicating specific amplification of the DNA sequence as in prior validation ([(Axelsson et al. 2020; Fitzpatrick et al. 2021; Serén et al. 2023)](https://paperpile.com/c/51ncpv/RLacJ+bCToQ+Mglbn)) |
| **Reaction mix & cycling conditions** | Fully detailed in Supplementary Methods |
| **Instrument details** | ThermoFisher QuantStudio Flex qPCR system specified |
| **PCR efficiency** | Calibrator-based relative qPCR design using LinRegPCR efficiency estimation instead of standard curve efficiency. Plate efficiencies reported (84.3% SCG, 88.7% telomere) |
| **Standard curves** | Calibrator-based relative qPCR design using LinRegPCR efficiency estimation instead of standard curve efficiency. Gold standard DNA used for calibration. |
| **Efficiency correction** | LinRegPCR applied; Pfaffl equation used |
| **Replicates** | Triplicate reactions per sample, samples outside of 0.5 Ct difference are excluded from downstream analysis. |
| **Controls** | “Gold standard” pooled DNA and no-template negative controls included on each plate. |
| **Dynamic range & detection limits** | – |
| **Inter-plate calibration** | Calibrator-based relative qPCR design using LinRegPCR efficiency estimation instead of standard curve efficiency. Gold standard DNA used for calibration. We have not ran inter-run variability assessment in this instance |
| **Raw Cq values** | Raw Cq values are found in the GitHub repository. |
| **Prediction intervals** | – |
| **Randomization / blinding** | Samples were not randomised in this study. |
| **Data normalization** | z-score transformation described |
| **Statistical analysis** | GLMMs, AICc model selection, hierarchical partitioning, cross-correlation with climate |
| **Reporting of effect sizes & CI** | Effect sizes, confidence intervals, model fit metrics provided |
| **Ethics & permits** | Fully documented in the main text |
| **Data availability** | GitHub link provided |

**Supplementary Table 2.** Sample sizes for each variable used in analyses.

| **Variable** | **Category** | **Sample size** |
| --- | --- | --- |
| Year | 2013 | 5 |
| Year | 2014 | 12 |
| Year | 2016 | 11 |
| Year | 2017 | 36 |
| Year | 2018 | 29 |
| Year | 2021 | 53 |
| Year | 2022 | 76 |
| Environmental type | A | 54 |
| Environmental type | B | 31 |
| Environmental type | C | 57 |
| Environmental type | D | 74 |
| Environmental type | RA | 6 |
| Sex | Female | 95 |
| Sex | Male | 95 |
| Sex | Unidentified | 32 |
| Morphotype (subspecies) | *G. g. eisentrauti* | 57 |
| Morphotype (subspecies) | *G. g. galloti* | 159 |
| Morphotype (subspecies) | *G. g. insulanagae* | 6 |
| Tissue type | Blood | 111 |
| Tissue type | Tail | 111 |

**Supplementary Table 3.** Sample sizes for each locality sampled (across years).

| **Locality code** | **Locality** | **Sample Size (across years)** |
| --- | --- | --- |
| BT | Base of Teide | 23 |
| CR | Cruz de Tea | 11 |
| CT | Cone of Teide | 21 |
| EM | El Medano | 26 |
| EP | El Pris | 10 |
| GR | Granadilla | 22 |
| IO | Izana Observatory | 12 |
| LG | La Guancha | 16 |
| LP | Llanito Perera | 10 |
| LR | Los Realejo | 9 |
| PA | Poris de Abona | 6 |
| PH | Punta del Hidalgo | 12 |
| RA | Roque de Anaga | 6 |
| RQ | Recibo quemado | 6 |
| TRE | Teide Refuge | 6 |
| TSE | Teide Slope | 6 |
| VL | Vilaflor | 20 |

**Locality as a random effect**

To account for potential spatial non-independence, we fitted models including locality as an additional random intercept. Including locality as a random effect explained negligible additional variance in telomere length (σ² ≈ 0), and did not alter inference for any fixed effects. Consequently, locality was not retained in the final models.

**Tissue type sensitivity analysis**

To assess whether tissue type influenced results, we fitted GLMMs separately for blood-only (n = 84) and tail-only (n = 103) datasets, and a model including tissue x Year interactions. Tail-only models reproduced the main patterns of the global analysis, with SVL remaining significantly negatively associated with TL (β = −0.129, *p* = 0.0039) and Env_PC1 weakly positively associated (β = 0.057, *p* = 0.021). The effect of sex (males shorter) remained directionally consistent (β = −0.839, *p* = 0.071). Blood-only models showed effects in the same direction (SVL: β = −0.035; sexM: β = −0.693), but none reached statistical significance (all *p* > 0.13), consistent with reduced statistical power. Models including Tissue x Year interactions showed no systematic tissue-specific temporal effects. Only a single interaction term (tail x 2018) was significant (β = −0.244, *p* = 0.042), while all other interaction terms were non-significant or not estimable. Core effects of SVL (β = −0.066, *p* = 0.021) and tissue type (β = 0.267, *p* = 0.0012) remained significant. These results confirm that tissue type does not confound observed temporal or intrinsic patterns.

**Morphotype sensitivity analysis**

To assess whether subspecies/morphotype influenced results, we included morphotype as a fixed effect in the GLMM. We compared the rarely sampled *G. insulanagae* (n = 6, sampled only in 2014), with *G. g. galloti* (n = 159) and *G. g. eisentrauti* (n = 57). Morphotype did not significantly predict shifted z-score in the full model (*galloti* vs. *eisentrauti*: β = 0.008, *p* = 0.913), and effect estimates were unchanged when the rare *G. g. insulanagae* were excluded. Core effects remained unchanged, with SVL negatively associated with telomere length (β = −0.088, *p* = 0.0030) and males exhibiting shorter telomeres than females (β = −0.682, *p* = 0.040). Environmental PCs remained non-significant (both *p* > 0.39). These results confirm that temporal and intrinsic patterns are robust to variation in subspecies representation.

**Removal of unidentified sexes**

To assess whether morphologically ambiguous individuals influenced the observed sex effect, we refitted the GLMM after removing individuals of unidentified sex (n = 32), leaving 175 individuals. Core effects remained unchanged: SVL was negatively associated with telomere length (β = −0.082, *p* = 0.0048), males had shorter telomeres than females (β = −0.874, *p* = 0.0074), and the SVL x sex interaction remained significant (β = 0.075, *p* = 0.025). Tissue type remained significant (β = 0.186, *p* = 0.00053), while environmental PCs remained non-significant (both *p* > 0.20). Estimated marginal means confirmed that females exhibited approximately 14% longer telomeres than males (ratio = 1.14, *p* = 0.0055). These results indicate that inclusion of unidentified sexes did not alter the primary conclusions, and their retention increases transparency of the full dataset.

**Additional model selection (Year as a fixed effect)**

Minimum, maximum and mean temperature, relative humidity, wind speed, solar radiation, radiant sky temperature, elevation, environment type, year, sex, snout-vent length (SVL) and tissue type were checked for collinearity using *corrplot* [(Wei and Simko 2024)](https://paperpile.com/c/51ncpv/ZHUJv) in R. All temperature variables collinear with elevation (>0.9) were removed, while radiant sky temperature (collinear with elevation) was retained due to its relationship with physiological change across elevation in *G. galloti* [(Gilbert et al. 2024b)](https://paperpile.com/c/51ncpv/spawA).

The rTL ratio was converted to a z-score from standardisation by subtracting the global mean value, and dividing by the standard deviation, according to Verhulst [(2020)](https://paperpile.com/c/51ncpv/6UhLD/?noauthor=1). A generalised linear model (GLM) was fitted with a Gamma distribution and log-link function, requiring a transformation (+1) to ensure positivity. Samples with missing data were removed.

The performance of models with all possible combinations of the predictors: year, environment type, elevation, mean relative humidity, mean wind speed, mean solar radiation, mean radiant sky temperature, morphotype (subspecies), tissue type, sex, and SVL was compared using the exhaustive heuristic algorithm (method = ‘*h*’) in the R package *glmulti* [(Calcagno and de Mazancourt 2010)](https://paperpile.com/c/51ncpv/HiOVf). Model selection was run without interaction terms, however two-way interactions were included between sex and SVL to test for sexual dimorphism and between year and tissue type to test for methods of tissue collection across years. To validate variable inclusion for the final model, predictors were checked for recurrence in the top 100 weighted models, revealing sex, year, and SVL occurred in 99% of models, followed by tissue type, and radiant sky temperature. The criterion for model fit to data used was the corrected-Akaike Information Criterion ‘*aicc*’, with the fit function ‘*glm*’ and confsetsize ‘100’ (size of returned confidence set).

**Model interaction**

Interaction terms between sex and SVL were included due to anticipated sexual size dimorphism, and tissue type with year due to differing collection methods across years. Both of these interaction terms were rejected during model selection, however the sex and SVL interaction was further investigated comparing models of deviance explained and AICc.

Deviance explained was calculated as:

$Deviance explained = 1 -\frac{Intrinsic/extrinsic}{Null deviance}$ .


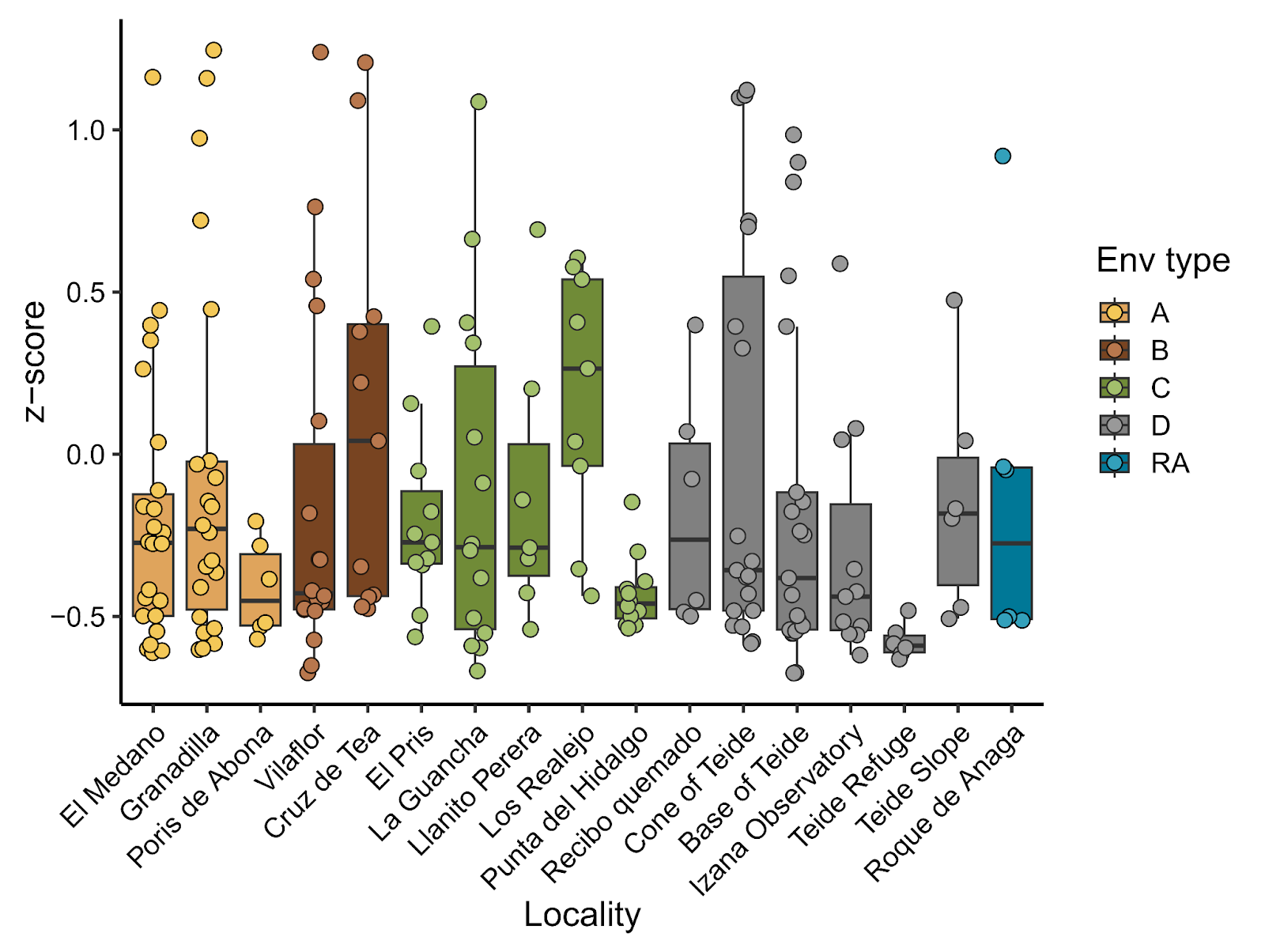


**Supplementary Figure 1.** Relative telomere length (transformed to z-scores) across different localities. Raw data, where each point is a single animal, is coloured by the environment type. Roque de Anaga (RA) is considered separately as an islet in the NE, occupied by the understudied *G. g. insulanagae* subspecies.

**Supplementary Table 4.** Variance explained and cumulative variance for each principal component.

| **Principal Component** | **Variance Explained** | **Cumulative Variance** |
| --- | --- | --- |
| 1 | 0.66 | 0.66 |
| 2 | 0.19 | 0.86 |
| 3 | 0.12 | 0.97 |
| 4 | 0.019 | 0.99 |
| 5 | 0.004 | 1 |

**Supplementary Table 5.** Raw loading contribution of each environmental variable, and calculated percentage contribution.

| **Environmental Variable** | **PC1 (66%)** | **PC2 (19%)** | **PC3 (12%)** | **PC4 (1.9%)** | **PC5 (0.4%)** |
| --- | --- | --- | --- | --- | --- |
| Elevation | -0.521 (27.1%) | 0.093 (0.9%) | 0.345 (11.9%) | 0.434 (18.9%) | -0.642 (41.2%) |
| Relative Humidity | -0.525 (27.5%) | 0.160 (2.6%) | 0.022 (0.0%) | -0.83 (68.8%) | -0.1 (1.0%) |
| Mean Windspeed | 0.395 (15.6%) | 0.138 (1.9%) | 0.884 (78.2%) | -0.204 (4.2%) | 0.036 (0.1%) |
| Solar Radiation | 0.112 (1.3%) | 0.971 (94.3%) | -0.178 (3.2%) | 0.108 (1.2%) | 0.028 (0.1%) |
| Radiant Sky Temperature | 0.533 (28.4%) | -0.059 (0.3%) | -0.259 (6.7%) | -0.264 (7.0%) | -0.759 (57.5%) |

**Supplementary Table 6.** Top three models. Models are ranked by AICc; all include a random intercept for year.

| **Model predictors (fixed effects)** | **df** | **logLik** | **AICc** | **ΔAICc** | **Weight** |
| --- | --- | --- | --- | --- | --- |
| Env_PC1 + sex + SVL + tissue type | 8 | −78.37 | 173.56 | 0.00 | 0.42 |
| sex + SVL + tissue type | 7 | −79.56 | 173.75 | 0.19 | 0.38 |
| morphotype + sex + SVL + tissue type | 8 | −79.15 | 175.11 | 1.55 | 0.19 |

**Supplementary Table 7.** Model fit of pseudo R^2^ (Nagelkerke) and deviance explained for the full model, a model with just the intrinsic variables, and just the external variables.

| **Model** | **Predictors** | **Deviance explained** | **Marginal R²** | **Conditional R²** |
| --- | --- | --- | --- | --- |
| Full | Intrinsic + extrinsic | 0.215 | 0.304 | 0.336 |
| Intrinsic | Tissue type, sex, SVL | 0.211 | 0.249 | 0.319 |
| Extrinsic | Environmental PCs | 0.004 | 0.005 | 0.164 |

**Supplementary Table 8.** Alternative generalised linear model including year as a fixed effect and all environmental variables after model selection. Shown are final model variables, estimates, standard error, t-value, p-value, and significance.

|  | **Estimate** | **Std. Error** | **t-value** | **p-value** | **Significance** |
| --- | --- | --- | --- | --- | --- |
| Intercept | -46.920 | 20.686 | -2.268 | 0.024 | ***** |
| Year | 0.024 | 0.010 | 2.308 | 0.022 | * |
| Tissue type: tail | 0.093 | 0.050 | 1.860 | 0.064 | NS |
| Sex: M | -0.164 | 0.050 | -3.283 | 0.001 | ****** |
| Sex: U | -0.126 | 0.084 | -1.503 | 0.134 | NS |
| SVL | -0.039 | 0.017 | -2.352 | 0.019 | ***** |
| Sky temp. | 0.008 | 0.004 | 2.218 | 0.028 | * |

**
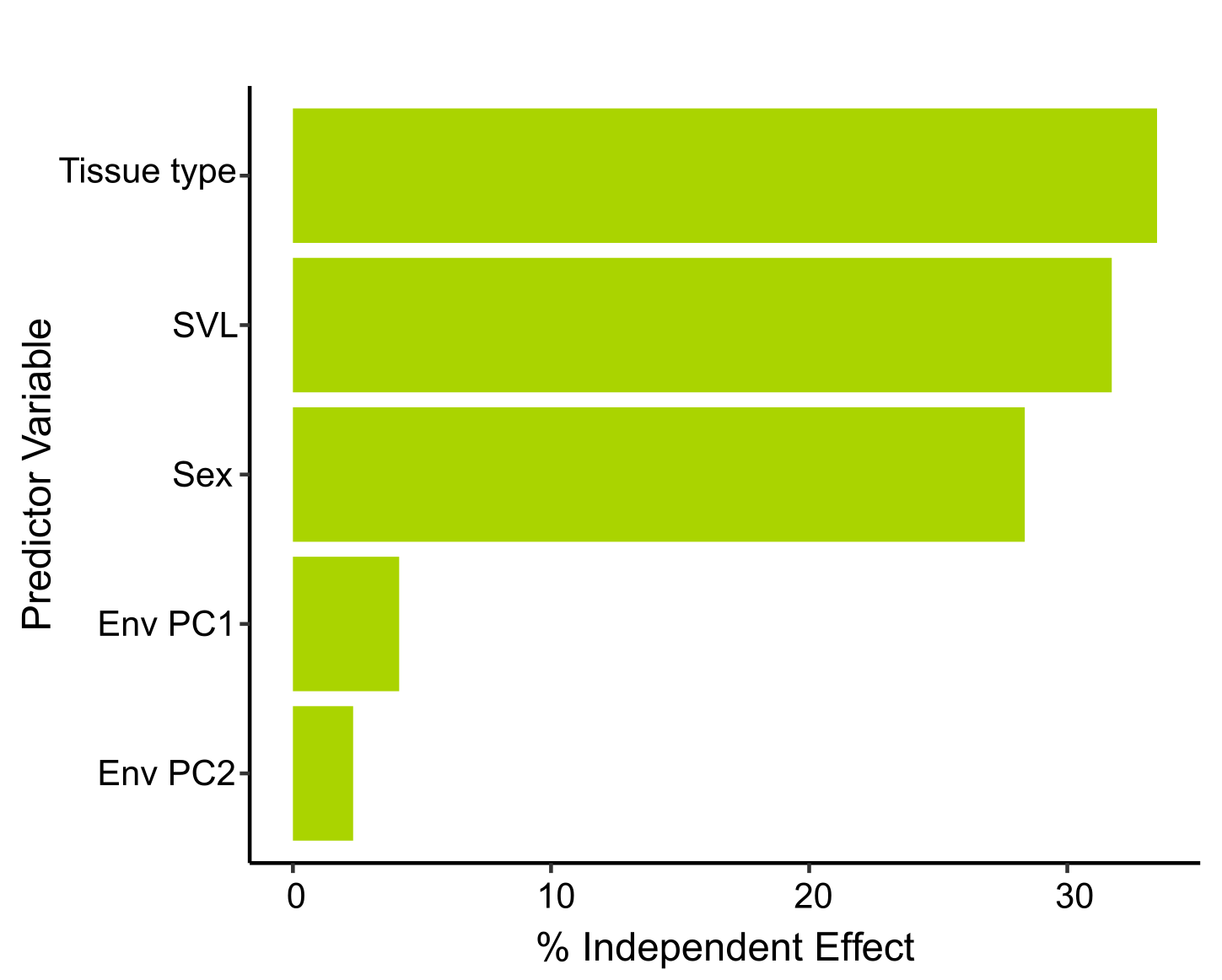
**

**Supplementary Figure 2.** Percentage of independent effects of predictors included in model on rTL, calculated using hierarchical partitioning. Independent effects are expressed as a percentage of the total explained variance.

**Supplementary Table 9.** Randomisation test of hierarchical partitioning examining statistical significance of the independent contributions of each predictor variable to the log-likelihood goodness-of-fit.

| **Predictor** | **Observed Contribution** | **Z-score** | **Significance (95%)** |
| --- | --- | --- | --- |
| Env PC1 | 1.2 | 1.32 |  |
| Env PC2 | 0.63 | -0.01 |  |
| Tissue type | 8.96 | 11.57 | * |
| Sex | 7.49 | 6.38 | * |
| SVL | 8.31 | 9.64 | * |
